# Pathogenesis of Cerebral Small Vessel Disease: Role of the Glymphatic System Dysfunction

**DOI:** 10.3390/ijms25168752

**Published:** 2024-08-11

**Authors:** Dong-Hun Lee, Eun Chae Lee, Sang-Won Park, Ji Young Lee, Man Ryul Lee, Jae Sang Oh

**Affiliations:** 1 Industry-Academic Cooperation Foundation, The Catholic University of Korea, 222, Banpo-daro, Seocho-gu, Seoul 06591, Republic of Korea; 2 Department of Medical Life Sciences, College of Medicine, The Catholic University of Korea, Seoul 06591, Republic of Korea; 3 Department of Neurosurgery, Uijeongbu St. Mary’s Hospital, College of Medicine, 271 Cheonbo-ro, Uijeongbu 11765, Republic of Korea; 4 Soonchunhyang Institute of Medi-bio Science (SIMS), Soon Chun Hyang University, Cheonan 31151, Republic of Korea

**Keywords:** cerebral small vessel disease, glymphatic system, magnetic resonance imaging, Virchow–Robin space

## Abstract

Cerebral small vessel disease (CSVD) is a group of pathologies that affect the cerebral blood vessels. CSVD accounts for 25% of strokes and contributes to 45% of dementia. However, the pathogenesis of CSVD remains unclear, involving a variety of complex mechanisms. CSVD may result from dysfunction in the glymphatic system (GS). The GS contains aquaporin-4 (AQP-4), which is in the perivascular space, at the endfeet of the astrocyte. The GS contributes to the removal of waste products from the central nervous system, occupying perivascular spaces and regulating the exchange and movement of cerebrospinal fluid and interstitial fluid. The GS involves astrocytes and aquaporin channels, which are components of the blood–brain barrier, and problems with them may constitute the pathogenesis of CSVD. Vascular risk factors, including diabetes, dilate the perivascular space, disrupting the glymphatic system and the active regulation of AQP-4. CSVD exacerbation due to disorders of the GS is associated with multiple vasculopathies. Dysfunction of the glymphatic system and AQP-4 interferes with the functioning of the blood–brain barrier, which exacerbates CSVD. In a long-term follow-up of CSVD patients with microbleeds, lacunar infarcts, and white matter hyperintensity, several vascular risk factors, including hypertension, increased the risk of ischemic stroke. Dysfunction of the GS may be the cause of CSVD; however, the underlying treatment needs to be studied further.

## 1. Introduction

Cerebral small vessel disease (CSVD) refers to several pathological diseases that affect blood vessels, including large to small blood vessels in the cerebrum [1]. Some imaging tests have shown that CSVD is directly related to the occurrence of Alzheimer’s disease (AD) and cerebrovascular disease. Key clinical symptoms based on several pathological and neurological processes in CSVD include stroke, cognitive decline, and dementia, accounting for 30% of ischemic stroke and cerebral hemorrhage cases [2]. In addition, other studies have identified higher cases of dementia in CSVD [3]. The prevalence of CSVD increases with age, affecting about 5% of the population aged 50 and 100% of the population aged 90 or older [3].

Recent studies suggest that vascular-related pathological diseases and the resulting cognitive impairment caused by these CSVDs are associated with glymphatic system (GS) dysfunction in the central nervous system (CNS) [4]. The GS is a system for removing waste from the CNS [5]. Dysfunction of the GS can contribute to CSVD, which in turn can lead to cognitive dysfunction.

However, GS dysfunction in CSVD has not been well studied. There is a lack of research on clear targets to improve the dysfunction of GS and a lack of research on how to treat it. Because the underlying mechanism of the disorder is unknown, current treatments for CSVD rely on symptomatic therapies such as antiplatelet and antithrombotic drugs. It is important to understand the pathogenesis of GS in CSVD to target treatment of the underlying cause.

## 2. Ischemic Cognitive Impairment on CSVD

CSVD is exacerbated by impairment of the neurovascular unit function, blood–brain barrier (BBB) damage, neuroinflammation, protein imbalance, and decreased blood flow [6]. CSVD is diagnosed based on neuroimaging marker detection such as cerebral atrophy, white matter hyperintensities (WMH), cerebral perforation, and cerebral microbleeds (CMB).

Two of the most common pathological changes associated with CSVD are arteriosclerosis and cerebral small vessel atherosclerosis [7]. Arteriosclerosis is the most common change in the aging brain. It causes small vessel atherosclerosis in the brain, causing thickening of blood vessel walls, loss of smooth muscle cells, and degeneration of internal elastic layers [8]. Ultimately, autoregulation of the associated small vessel disease is impaired, resulting in chronic cerebral hypovascularization of cerebral blood flow (CBF) [9].

CSVD leads to a decrease in brain blood flow, leading to cerebral ischemia. Cerebral ischemia is a condition in which blood flow to the brain is insufficient to meet metabolic needs. Cerebral ischemic conditions can lead to a lack of oxygen supply or hypoxia, leading to ischemic stroke due to the death of brain tissue [10]. During brain ischemia, the brain cannot perform aerobic metabolism due to loss of oxygen and substrate and ATP levels drop sharply. A lack of energy causes cells to lose their ability to maintain electrochemical gradients, resulting in mass release of glutamate from synaptic vesicles, high concentration of calcium in the cytoplasm, suspension of protein synthesis, and brain cell death due to metabolic waste removal disorders.

CSVD is also thought to result in dysfunction of the BBB. The BBB is a selective semipermeable border membrane of endothelial cells [11]. It allows the passage of some small molecules by diffusion in the CNS, where neurons are located, and selective active transport for nutrients and macromolecules necessary for neural function. The BBB protects the brain by preventing peripheral immune factors, such as antibodies or immune cells, from entering the CNS. CSVD results in BBB dysfunction that stems from ischemic stroke and metabolic dysfunction within the brain. In several radiological studies, intravascular neointima into brain tissue with BBB dysfunction has been reported in patients with small vessel disease (SVD) [12].

Ischemic conditions and BBB dysfunction lead to cerebrovascular events such as stroke or vascular cognitive impairment (VCI). The VCI is a condition in which cerebrovascular disease contributes to a decline in mental performance [13]. CSVD is the most prevalent of several mechanisms associated with VCI [14]. A clinical study of patients with CSVD found that CSVD patients performed worse than healthy controls on cognitive assessments such as the mini-mental state examination (MMSE) and Montreal cognitive assessment (MoCA) [15].

## 3. Glymphatic System in CNS

CSVD induces BBB dysfunction. Recent research suggests a relationship between BBB dysfunction and GS dysfunction on CSVD. GS means the glial and lymphatic system, namely the lymphatic system in the CNS. In the GS, cerebrospinal fluid (CSF) is circulated [16]. The CSF is a clear fluid found in the tissues surrounding the brain and spinal cord. The CSF escapes through the perivascular space (PVS) around the cerebral arteries. This CSF is circulated in the GS. The GS is a system for waste removal from the vertebrate CNS. Astrocytes branch out like branches to form endfeet, which are the main components of the BBB. The astrocytes spread into blood vessels and line the outer layer of capillaries.

Direct evidence has been proposed that the GS increases interstitial waste removal during the resting state [17]. To facilitate the clearance of interstitial wastes such as amyloid beta, expansion and contraction of the extracellular space occurred, which increased by 60% in the sleeping brain.

Several key functions of the GS have been demonstrated, with the brain’s peri-vascular pathways playing a key role [18]. In this system, CSF and ISF combine to flow into the PVS around the cerebral arteries and exit into the perivenous space around the cerebral arteries and exit into the perivenous space [5]. Interstitial fluid (ISF) is similar in nature to plasma. ISF is the fluid between blood vessels and cells, which transports waste products released by cells due to metabolism [19].

### 3.1. Perivascular Space

**Figure 1:**
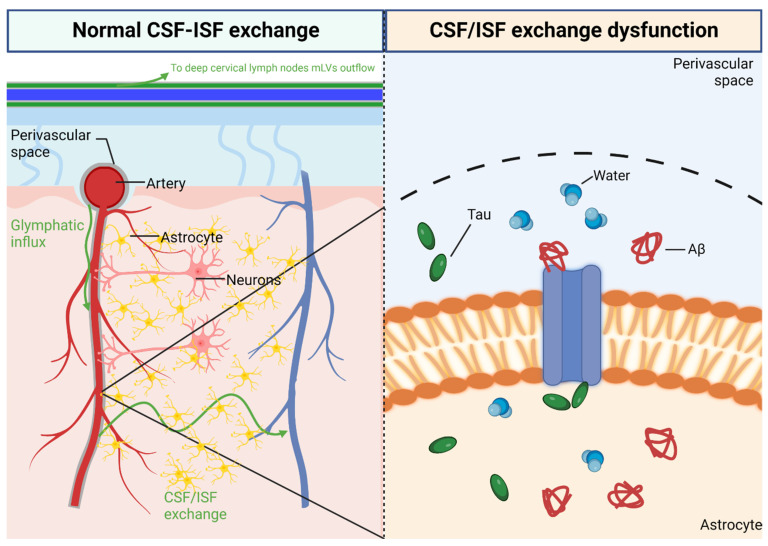
In the perivascular space surrounding the artery, there are numerous endfeet of astrocytes. There are many AQP-4 channel proteins there, which are responsible for water transport. CSF: cerebrospinal fluid, ISF: interstitial fluid.

The PVS is the space surrounding blood vessels in the brain and other organs and has an immunologic function [20]. The PVS constitutes extravascular channels that allow blood vessels to penetrate and communicate with the brain and other organs (shown in Figure 1) [21].

The PVS, like the blood vessels that form around them, are found in the subarachnoid space and subpial space of the brain. One of the most basic roles of the PVS is to regulate fluid movement and drainage in the CNS. The PVS drains fluid from nerve cell bodies to the cervical lymph nodes [21]. The PVS is an integral part of the BBB.

Cellular debris and foreign bodies, which are often impermeable to the BBB, pass through the endothelial cells and phagocytize only in the PVS. This applies to many T cells and B cells as well as monocytes, giving this small fluid-filled space an important immunologic role [22].

### 3.2. Aquaporin Channel

Aquaporins (AQP) are channel proteins that form pores in the membranes of cells, primarily facilitating water transport between cells [23]. The CNS described above is produced in the choroid plexus, which is assisted by AQP-1. AQP-1 is expressed on the membrane of the choroid plexus [24]. Across AQP-1, CSF is transported into the cell [25]. The AQP-4 is an integral membrane protein that conducts water across cell membranes [26]. AQP-4 is most abundant in the cerebellum and spinal cord [27]. In the CNS, AQP-4 is the most prevalent AQP channel, specifically located at the endfeet of microvascular astrocytes and the endfeet of glial cells [28]. The AQP-4 is commonly found to facilitate water movement in the CNS [29]. AQP-4 may be involved in various physiological processes such as waste removal and potassium homeostasis through the GS [30]. The AQP-4 is essential for memory formation and synaptic plasticity [30]. The AQP-4 is upregulated by direct damage to the CNS [31]. The AQP channels are highly concentrated in the BBB [30]. 

## 4. Glymphatic System Dysfunction on CSVD

Dysfunction of the GS leads to exacerbation of CSVD, exacerbating cerebrovascular and neurodegenerative diseases. In a rat model with multiple microinfarctions, research found that the expression of AQP-4 was reduced [32]. Animal model studies suggest that AQP-4 plays pathological responses in AD, Parkinson’s, traumatic brain injury, and stroke [30]. The AQP-4 knockout mice exhibit cognitive problems, with impairments in memory acquisition and spatial recognition [30]. The AQP-4 is important in both the development and recovery from stroke and cerebral edema [33]. However, other clinical studies addressing the relationship between AQP-4 and GS have shown opposite results. When blood samples from acute stroke patients were analyzed by ELISA, the expression of AQP-4 was increased in the patient population compared to healthy controls [34]. Although the results were opposite to the preclinical studies described above, they found that AQP-4 was either increased or decreased depending on the patient’s condition. This will require further research.

Cerebral amyloid angiopathy is a vascular failure often associated with AD that uses ePVS to spread inflammation to the brain parenchyma. A report found a higher prevalence of ePVS in patients with AD [35]. It has been hypothesized that PVS structures in the cerebral cortex may contribute to the development of AD and a study supporting this hypothesis noted a higher frequency of amyloid beta plaques in the cerebral cortex than in the basal ganglia in patients with AD [36].

More widespread PVS was observed in a study of magnetic resonance imaging (MRI) scans of people diagnosed with multiple sclerosis [37]. A study of 1026 patients with brain MRIs from The Multi-Ethnic Study of Atherosclerosis (MESA) cohort, collected from six centers in the United States, found that larger basal ganglia and thalamic ePVS volumes were associated with a larger total WMH volume, lower WMFA, and higher odds for CMBs. A larger insular ePVS volume was associated with a smaller total WMH volume. Temporal ePVS volume was positively associated with total gray matter volume [38].

## 5. Risk Factors of Glymphatic System Dysfunction on CSVD

Exacerbation of GS leads to small vessel disease and induction of several types of CSVD. The prevalence of CSVD was associated with poor long-term outcomes for patients and was consistently associated with significantly increased mortality.

Studies examining risk factors in patients with CSVD have shown that image markers of CSVD such as CMB, WMH, and lacunar infarcts are associated with the risk of developing dangerous conditions such as stroke and Alzheimer’s disease. Hypertension, diabetes, hypercholesterolemia, and smoking have been consistently proven to be major risk factors for CSVD [39]. 

**Figure 2:**
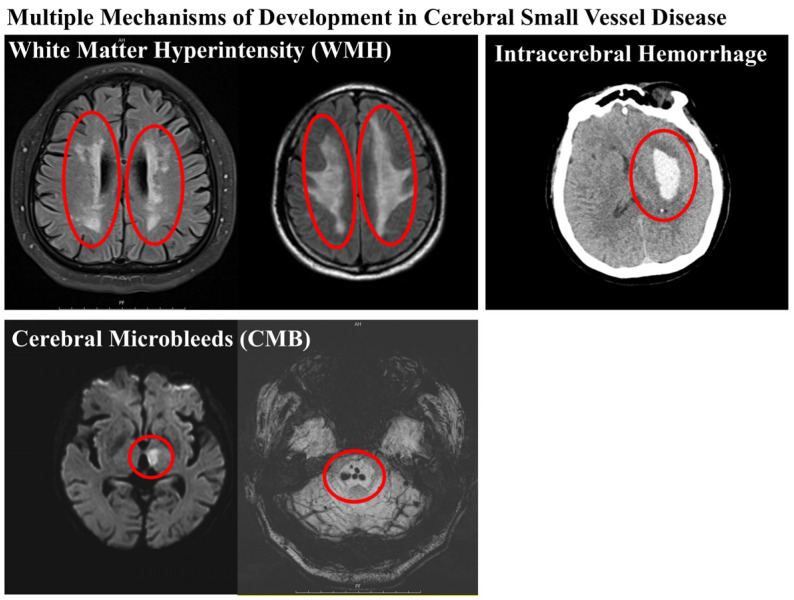
Brain MRI images of patients who have experienced CSVD. WMH, CMB, lacunar infarct, and ICH worsen CSVD due to ischemic or hemorrhagic lesions. The red circles within the brain are cerebral hemorrhage and ischemic conditions identified by WMH, CMB, infarct, and hemorrhage occurrence points, respectively.

Visualization studies of neuroimaging features of CSVD are a common method for identifying neurologic lesions [40]. According to the Standards for Reporting Vascular Changes in Neuroimaging (STRIVE), neuroimaging features of CSVD include recent small subcortical infarcts (RSSI), white matter hyperintensity (WMH), lacunes, PVS, CMB, and brain atrophy (shown in Figure 2) [41].

### 5.1. Cerebral Microbleeds

CMB is a prognostic marker to identify vascular events of underlying chronic small vessel disease. This disorder has a high prevalence in elderly patients and is particularly prevalent in patients with stroke, small blood vessel genetic disorders, and other vascular diseases. Furthermore, in our review of studies on CMB and CSVD, it was stated that deep CMB is generally thought to be associated with hypertension-related SVD (i.e., atherosclerosis) [42]. 

A meta-analysis of 31 cohort studies including data from 20,368 patients showed that in patients without stroke, patients with CMB had a significantly higher hazard ratio of first symptomatic intracerebral hemorrhage (sICH) and ischemic stroke (IS) [43]. Moreover, the location of the CMB was particularly important: lobar CMBs were associated with a significantly higher risk of sICH, whereas there was no association with the risk of IS. In contrast, deep CMBs were associated with a significantly higher risk of sICH and IS [44]. Additionally, in an analysis of data from 10 studies including 1306 patients, the presence of CMB was associated with an increased risk of recurrent intracranial hemorrhage (ICH) [45].

### 5.2. White Matter Hyperintensities

White matter hyperintensities (WMH) are white matter lesions that are brightly visible on fluid attenuation inversion recovery (FLAIR) MRI. These lesions are known to be a sign of CSVD [46]. Symptomatic development of WMH accompanies multiple physiologic and pathologic changes in ischemic lesions, loss and deformation of the myelin sheath, damage to the walls of small vessels, gliosis, micro-hemorrhages, and breaches of the cerebrospinal fluid (CSF), BBB [47]. 

### 5.3. Lacunar Infarct

Lacunar infarcts are a type of stroke that occurs when blood flow to a small area of brain tissue is blocked. Today, lacunar strokes, which are caused by occlusion of a single penetrating artery in a larger cerebral artery, account for up to 20–30% of all acute ischemic strokes [48]. 

Although these infarcts are small compared to those caused by large artery occlusions, they were found to be the most common type of acute stroke (37%) in the Harvard stroke registry study [49].

In research using MRIs of 43 patients with lacunar infarction, pure motor stroke was the most frequent finding (n = 20, 47%), followed by ataxic hemiparesis (n = 11, 26%), sensorimotor stroke (n = 9, 21%), dysarthria-clumsy hand syndrome (n = 2, 5%), and pure sensory stroke (n = 1, 2%) [50]. 

### 5.4. Long-Term Outcome of CSVD Patients

The research that determined the effect of vascular risk factors and baseline CSVD burden on progression in patients from 2006 to 2020 included 382 patients with CSVD who underwent at least two MRI scans with a mean (SD) follow-up of 11.15 (3.32) years. In the study, Assessment of Baseline CSVD Burden measured WMH volume, lacune count, and microbleed count at baseline as CSVD markers. In analysis, baseline age predicted WMH progression. There was also a significant association between WMH volume during 14 years of follow-up. The yearly mean (SD) progression rate in patients was 0.6 (0.74) mL/y (range, 0.02–4.73 mL/y). WMH progression was faster in the group with two or more concurrent vascular risk factors such as smoking, diabetes, hypercholesterolemia, and hypertension [51]. 

Additionally, we investigated research with up to 5 years of long-term follow-up of patients with acute cerebral infarction or transient ischemic attack enrolled between January 2009 and December 2010. In this research, the total CSVD score for each patient was calculated by calculating the presence or absence of CMB, WMH, EPVS, and lacunar infarction on an ordinal scale from 0 to 4. Furthermore, in multivariate COX regression analysis adjusting for age, sex, and variables in the paper, the presence of CMBs, WMH, or EPVS was independently associated with all-cause mortality. Specifically, CMB was associated with fatal hemorrhagic stroke and the total CSVD score was significantly associated with all-cause mortality, fatal ischemic stroke, and fatal hemorrhagic stroke [52].

## 6. Discussion

CSVD is an important contributor to vascular and age-related cognitive decline and dementia risk but treatment strategies are not well understood; essentially, CSVD is caused by universal vascular risk factors such as hypertension, arteriosclerosis, and brain damage from WMH and CMBs and the problems with GS described above can also exacerbate CSVD. Approaches to treating CSVD include targeting the cause of CSVD or implementing countermeasures that target the consequences of CSVD.

Pharmacologic treatments are available to improve existing cognitive impairment. Anticholinesterase (AChE)-selective inhibitors, such as donepezil, which is licensed for the treatment of AD, prevent the breakdown of the neurotransmitter acetylcholine. Phosphodiesterase-5 inhibitors can also be expected to improve vascular cognitive impairment; but, in cases such as CMB that are not due to ischemic damage, the disease may worsen. Anticoagulants and antiplatelet agents are also effective in cases such as stroke, but they have no reported use in CSVD and carry the risk of increased bleeding. Conversely, a chemical agent that may be used in bleeding conditions is endothelin-1. It is a potent peptide vasoconstrictor and there are reports of its use in the treatment of SAH. Rho-kinase (ROCK) inhibitors increase BBB integrity in stroke and vascular cognitive dysfunction [53]. Although not reported in SVD, complementation of BBB function may help improve glymphatic dysfunction.

However, other studies suggest that the pathophysiology of CSVD is complex and involves different pathways compared to the thromboembolic pathways that cause traditional ischemic stroke. Compromised BBB integrity due to GS problems is a concern and it is argued that vascular therapies, including antiplatelet drugs, may not be effective in CSVD. To improve GS, lifestyle modifications may help improve and prevent CSVD in the long term. Improving sleep quality is a possible way to improve glymphatic dysfunction [17]. The GS has been shown to be active during sleep and the removal of solutes such as amyloid-beta is slowed during wakefulness. Studies have consistently linked SVD to sleep disorders and one study found that PVS dilation correlated with sleep quality [54]. All of this suggests that as sleep quality declines, so does the function of the GS. Treatments targeted at improving sleep are popular, such as pharmacologic therapies such as sleeping pills, but there are no studies to confirm that they directly improve the GS. In addition, general health improvement factors such as a healthy diet, exercise, and smoking cessation, as well as improvements in vascular risk factors, can benefit the health of the entire body, including CSVD [55].

Vascular risk factors in CSVD can induce hemorrhagic or ischemic conditions, leading to the development of vascular lesions and cognitive impairment. In addition, GS dysfunction in the CNS leads to problems such as amyloid angiopathy due to impaired waste removal, which is also associated with dilation of the perivascular space and disruption of aquaporin channels. Clinically, stroke, infarction, CMB, WMH, etc., are image markers of CSVD and have been strongly associated with the disease. Several vascular risk factors are involved in CSVD. However, we need to look at the underlying causes from which these vascular risk factors themselves come.

In order to prevent and treat these CSVDs, lifestyle modification is required along with existing vascular risk factors. Common risk factors such as hypertension and irregular sleep patterns have both been associated with CSVD and measures to improve these may help improve CSVD. In addition, conventional pharmacological treatments such as antiplatelet agents to treat bleeding or ischemic conditions, although not reported in patients with CSVD, can be suggested as a treatment modality. Several pharmacologic treatments have been proposed but none have been reported in patients with CSVD. These suggestions will require continued follow-up studies.

There are not many preclinical studies to investigate the relationship between CSVD and GS. It may be difficult to replicate the dysfunction of GS at the animal or cellular level. Also, GS is a relatively new concept and there is not much research on it. These points need to be addressed for continued follow-up studies.

## 7. Conclusions

CSVD is significantly influenced by glymphatic system (GS) dysfunction, which can lead to impaired waste removal and amyloid angiopathy, contributing to vascular lesions and cognitive impairment. Preventing and treating CSVD requires addressing both traditional vascular risk factors and GS-related issues. Lifestyle modifications, such as improving sleep quality and managing hypertension, are essential. While conventional pharmacological treatments like antiplatelet agents have not been widely reported for CSVD, they could be considered alongside new approaches targeting GS dysfunction. Continued follow-up studies are necessary to validate these treatment strategies in CSVD patients.

## Data Availability

Not applicable.
